# Effect of SGLT2 inhibitors on microscopic hematuria in IgA nephropathy: a review from the perspective of disease activity biomarkers

**DOI:** 10.3389/fmed.2026.1834859

**Published:** 2026-05-29

**Authors:** Junhua Yang, Zesheng Chen, Haochuan Han, Can Li, Dafeng Yue

**Affiliations:** 1 Department of Nephrology, Ziyang Central Hospital, Ziyang, Sichuan, China; 2 Department of Medical Imaging, Ziyang Central Hospital, Ziyang, Sichuan, China

**Keywords:** disease activity biomarker, glomerulonephritis, IgA nephropathy, microscopic hematuria, renoprotection, SGLT2 inhibitors

## Abstract

IgA nephropathy (IgAN) is the most common primary glomerulonephritis worldwide. Microscopic hematuria, which is thought to reflect glomerular capillary wall injury but cannot by itself distinguish active inflammation from chronic structural damage, has recently been re-recognized as an independent predictor of disease progression in IgAN, yet it has long been overlooked in the assessment of treatment response. SGLT2 inhibitors have been demonstrated to reduce proteinuria and slow the decline of kidney function in patients with IgAN and are now incorporated into first-line supportive therapy. However, existing clinical trials and guidelines have focused almost exclusively on proteinuria and estimated glomerular filtration rate (eGFR), and the effect of SGLT2 inhibitors on microscopic hematuria has not been systematically evaluated. From the perspective of disease activity biomarkers, this review summarizes the pathophysiological basis and prognostic value of hematuria in IgAN, analyzes the hemodynamic, anti-inflammatory, and cytoprotective mechanisms through which SGLT2 inhibitors may influence hematuria, critically appraises the limited clinical evidence currently available, and compares these findings with hematuria data from novel targeted therapies including anti-APRIL monoclonal antibodies and complement inhibitors. This review aims to identify a key evidence gap in the current therapeutic framework of IgAN and to frame the effect of SGLT2 inhibitors on hematuria as an important but insufficiently studied question that warrants dedicated prospective investigation.

## 1 Introduction

Immunoglobulin A nephropathy (IgA nephropathy, IgAN) is the most common primary glomerulonephritis worldwide, with a particularly high prevalence in the Asia-Pacific region and significant geographic and ethnic variations (1–3). The pathogenesis of IgAN can be elucidated through the “four-hit hypothesis”: mucosal immune dysregulation leads to the overproduction of galactose-deficient IgA1 (Gd-IgA1), which subsequently induces autoantibody generation and pathogenic immune complex formation, ultimately depositing in the glomerular mesangium and triggering complement activation and inflammatory injury (4–7). This immune-mediated glomerular injury manifests clinically as hematuria and proteinuria (8). Although the establishment of the Oxford MEST-C classification (9) and the International IgAN Risk Prediction Tool (10) has improved risk stratification, approximately 30%−40% of patients still progress to end-stage kidney disease (ESKD) within 20 years of diagnosis (11–13), and long-term outcomes remain suboptimal even under contemporary treatment strategies (14). Supportive therapy centered on renin–angiotensin system inhibitor (RASi) therapy remains the current cornerstone of management (15), but a considerable proportion of patients continue to experience progressive kidney function decline despite maximized therapy, underscoring the need for additional therapeutic options.

Proteinuria has long been regarded as the most reliable predictor of disease progression in IgAN; however, microscopic hematuria, which has been proposed as a candidate marker of glomerular inflammatory activity, is receiving increasing attention. Hematuria is generally thought to indicate injury to the glomerular capillary wall, although it cannot reliably distinguish active inflammation from chronic vascular or structural lesions, and its mechanistic origin is considered at least partly distinct from that of proteinuria—the latter may also arise from chronic structural changes such as glomerulosclerosis. In a landmark study, Sevillano et al. (16) demonstrated that remission of hematuria was independently associated with improved kidney survival, and an accompanying editorial described hematuria as “the almost forgotten biomarker” (17). Subsequent meta-analyses confirmed that persistent hematuria is a significant risk factor for kidney disease progression and ESKD (18); even in patients with mild proteinuria and preserved kidney function, hematuria was associated with a higher risk of progression (19); and long-term outcomes in patients with recurrent episodes of gross hematuria were also unfavorable (20). Zand et al. (21) advocated for the incorporation of microscopic hematuria into the monitoring protocols of patients with IgAN, and a comprehensive biomarker review also positioned hematuria as a clinically actionable indicator of disease progression (22). Floege et al. (23) formally proposed that microscopic hematuria can serve as a biomarker of disease activity in IgAN, while noting limitations such as the lack of standardized measurement methods and the inability of hematuria alone to distinguish between inflammatory and chronic lesions.

Sodium-glucose cotransporter 2 (SGLT2) inhibitors represent a major breakthrough in kidney protection in recent years. This class of drugs reduces intraglomerular pressure by restoring tubuloglomerular feedback (24) and exerts multiple protective mechanisms including anti-inflammatory and antifibrotic effects (25, 26). Landmark trials such as CREDENCE (27), DAPA-CKD (28), EMPA-KIDNEY (29), and DIAMOND (30) have established the therapeutic role of SGLT2 inhibitors in chronic kidney disease (CKD). In the field of IgAN, a prespecified subgroup analysis of DAPA-CKD demonstrated that dapagliflozin significantly reduced the incidence of major adverse kidney events (31), and a secondary analysis of EMPA-KIDNEY also suggested a favorable trend with empagliflozin in the glomerular disease subgroup (32). Based on this evidence, SGLT2 inhibitors have been regarded as a potential new standard of care in IgAN (33), and the 2025 KDIGO guidelines have incorporated them into the first-line supportive therapy framework.

However, the existing evidence has focused almost exclusively on proteinuria and eGFR as outcome measures. Whether SGLT2 inhibitors also affect microscopic hematuria—a marker increasingly recognized as reflecting glomerular inflammation—has not been systematically evaluated. To date, only one observational study has reported a significant reduction in hematuria in patients with IgAN after 6 months of SGLT2 inhibitor therapy (34), and hematuria was documented only as a secondary observational variable. No previous review has addressed this topic in a dedicated manner.

**Figure 1:**
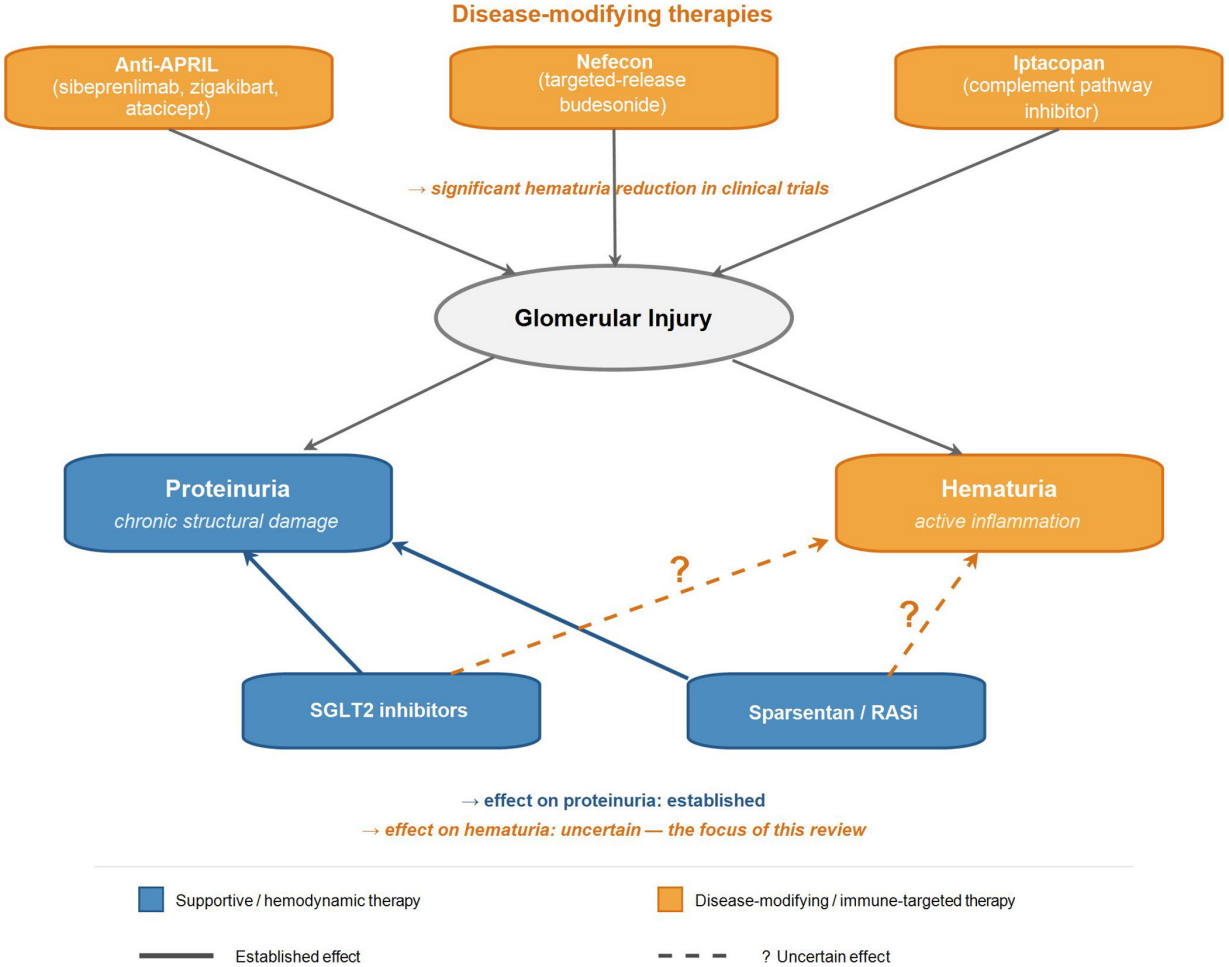
Treatment targets and hematuria in IgA nephropathy: supportive vs. disease-modifying therapies.

This narrative review aims to delineate this evidence gap rather than to resolve it. Its primary objective is to map the currently available evidence—direct, indirect, and mechanistic—bearing on whether SGLT2 inhibitors affect microscopic hematuria in patients with IgAN, and to clarify where the evidence ends and where hypothesis begins. Additionally, this review explores whether changes in hematuria might, hypothetically, reflect anti-inflammatory effects beyond hemodynamic mechanisms, and places these tentative inferences alongside hematuria data reported in trials of novel disease-modifying therapies, including anti-APRIL monoclonal antibodies, complement inhibitors, and targeted-release budesonide. By integrating the above evidence, this review seeks to motivate the design of future clinical trials that would formally test hematuria as a treatment response endpoint. The overall conceptual framework of this review - contrasting the established effect of supportive/hemodynamic therapies on proteinuria with the more uncertain effect of these agents on hematuria, and juxtaposing this with the hematuria reductions reported for disease-modifying therapies - is summarized in Figure 1. Whether hematuria should ultimately enter routine therapeutic monitoring of IgAN is a question that must await such prospective validation. Consistent with this narrative format, a focused (non-systematic) literature search was performed in PubMed, Embase, and Web of Science using the terms “IgA nephropathy,” “SGLT2 inhibitors,” and “hematuria,” with a cutoff date of January 2026, supplemented by manual screening of reference lists. Studies were selected by relevance to the review's aims rather than by predefined inclusion or exclusion criteria, and no formal quality appraisal or PRISMA-style workflow was applied; the present article should therefore be read as a narrative review with a focused literature search, not as a systematic review.

## 2 Microscopic hematuria as a biomarker of disease activity in IgAN

### 2.1 Pathophysiological basis of hematuria

Microscopic hematuria in IgAN is not merely a non-specific urinary abnormality; rather, it is thought to arise, at least in part, from immune-inflammatory injury to the glomerular capillary wall. According to the prevailing model, when IgA immune complexes deposit in the mesangium and activate the complement cascade, the release of inflammatory mediators disrupts the integrity of the glomerular basement membrane and endothelial cell layer, allowing erythrocytes to leak through the damaged capillary wall into the urine (17). This process corresponds pathologically to the endocapillary hypercellularity (E1) and crescent formation (C1/C2) scores in the Oxford MEST-C classification—both of which reflect active glomerular inflammation and are positively correlated with the severity of microscopic hematuria (23). It should be emphasized at the outset, however, that this mechanistic framework represents one plausible interpretation of a heterogeneous biomarker; the same urinary finding may also arise from chronic vascular or structural lesions, and the extent to which a given patient's hematuria reflects ongoing inflammation cannot be determined from the urinalysis alone (see Section 2.3).

It is important to emphasize that although hematuria and proteinuria frequently coexist in patients with IgAN, they reflect distinct pathological processes. Proteinuria is more closely associated with chronic structural changes such as podocyte injury, glomerulosclerosis, whereas hematuria has been proposed to track more closely with ongoing glomerular inflammation, although, as noted below (Section 2.3), it cannot by itself distinguish inflammatory from chronic structural injury. This implies that in the same patient, proteinuria and hematuria can provide complementary information regarding disease status: proteinuria reflects the extent of cumulative damage, whereas hematuria may more sensitively capture ongoing inflammatory activity (21). Duan et al. (22), in a review of blood and urinary biomarkers in IgAN, also noted that hematuria, as a non-invasive, low-cost, and reproducible indicator, carries independent prognostic information distinct from proteinuria and should be incorporated into the comprehensive evaluation system for IgAN.

### 2.2 Prognostic value of hematuria

The association between hematuria and kidney outcomes in IgAN has been supported by robust evidence over the past decade. Sevillano et al. (16) followed 112 patients with IgAN for a median of 14 years and stratified them according to time-averaged hematuria levels into persistent hematuria and hematuria remission groups. The results demonstrated that patients with persistent hematuria had a significantly higher proportion of progression to ESKD or a 50% decline in kidney function compared with those who achieved hematuria remission. Multivariable analysis confirmed that time-averaged hematuria was an independent predictor of ESKD, and following the disappearance of hematuria, the rate of kidney function decline decreased sharply from −6.45 ml/min/ 1.73 m^2^ per year to −0.18 ml/min/1.73 m^2^ per year. This study also revealed an important finding: among patients with proteinuria >0.75 g/day, only those with concomitant persistent hematuria had significantly lower kidney survival compared with all other groups, suggesting that hematuria provides additional stratification value beyond the prognostic prediction afforded by proteinuria.

These findings have been validated in larger-scale studies. A systematic review and meta-analysis by He et al. (18) synthesized data from multiple studies, confirming that hematuria is a significant risk factor for kidney progression and ESKD in IgAN, and that this association is independent of proteinuria levels. Liu et al. (19) further focused on a low-risk population—patients with IgAN who had mild proteinuria and preserved kidney function—and found that even in this subgroup traditionally considered to have a favorable prognosis, persistent hematuria was associated with a more rapid rate of kidney function decline. For patients with recurrent episodes of gross hematuria, a long-term follow-up study by Le et al. (20) showed that although some patients had acceptable outcomes between episodes of gross hematuria, overall kidney outcomes exhibited significant heterogeneity, with some patients ultimately progressing to ESKD.

Regarding remission criteria, Japanese investigators proposed a composite definition of remission in IgAN, requiring simultaneous remission of both hematuria and proteinuria (urinary sediment erythrocytes <5/HPF and proteinuria <0.3 g/day) sustained for at least 6 months. Matsuzaki et al. (35) validated this criterion and found that patients who achieved simultaneous remission of both hematuria and proteinuria had the best kidney prognosis, those who achieved neither had the worst, and those who achieved remission of only proteinuria or only hematuria had intermediate outcomes. This result strongly supports the rationale for incorporating hematuria into the assessment of treatment response.

### 2.3 Potential and limitations of hematuria as a marker of treatment response

The above evidence suggests that microscopic hematuria has the potential to serve as a supplementary indicator for monitoring treatment response in IgAN, particularly in evaluating whether a drug effectively suppresses glomerular inflammatory activity. Coppo and Fervenza (17), in their editorial, called for renewed attention to the monitoring value of hematuria in both clinical trials and routine practice, rather than focusing exclusively on proteinuria and eGFR. Zand et al. (21) more explicitly advocated that hematuria should be incorporated into the screening and treatment monitoring protocols of patients with IgAN, serving as one of the bases for guiding therapeutic decisions.

However, the methodological challenges facing hematuria as a biomarker cannot be ignored. Floege et al. (36) systematically reviewed these limitations in their latest publication. Although urine dipstick testing is simple and inexpensive to perform, it detects hemoglobin (including free hemoglobin and myoglobin) rather than erythrocytes themselves, and therefore cannot distinguish true microscopic hematuria from hemoglobinuria. Urine sediment microscopy, while capable of directly counting erythrocytes, is subject to considerable variability due to specimen processing, centrifugation conditions, and inter-observer interpretation. Automated urine analyzers can partially overcome the subjectivity of manual interpretation, but the comparability between different instrument platforms has not been fully validated. This lack of uniformity in measurement methods directly contributes to difficulties in cross-study comparisons—definitions of hematuria severity and remission thresholds vary across clinical trials, posing challenges for data synthesis.

A more fundamental issue is that although hematuria suggests injury to the glomerular capillary wall, hematuria alone cannot distinguish between active inflammation (such as crescent formation and endocapillary hypercellularity) and chronic lesions (such as increased vascular fragility due to vascular sclerosis). Floege et al. (36) illustrated this point with a case report: a patient had a urinary sediment erythrocyte count of <3/HPF but a dipstick result of hemoglobin 1+, and repeat kidney biopsy confirmed crescentic IgAN. This suggests that clinicians, when interpreting hematuria results, need to consider them in the overall context of proteinuria levels, changes in kidney function, and the clinical course, and should consider repeat kidney biopsy when necessary to clarify disease activity.

Moreover, the evidence supporting hematuria as an independent predictor is not entirely uniform. Le et al. (20) reported substantial heterogeneity in long-term outcomes among patients with recurrent macroscopic hematuria, with some achieving favorable kidney survival despite episodic hematuria, indicating that the prognostic weight of hematuria varies with clinical context. Importantly, no prospective trial has used hematuria remission as a primary endpoint, and neither a validated remission threshold nor a minimum clinically important change in urinary erythrocyte count has been established—limitations that constrain the interpretability of any treatment-associated hematuria change reported to date.

## 3 Kidney-protective mechanisms of SGLT2 inhibitors: beyond hemodynamics

### 3.1 Tubuloglomerular feedback and hemodynamic effects

The kidney-protective effects of SGLT2 inhibitors were initially attributed to their modulation of kidney hemodynamics. Under physiological conditions, SGLT2 is located in the S1 segment of the proximal tubule and is responsible for reabsorbing approximately 90% of filtered glucose. When SGLT2 is inhibited, sodium and glucose reabsorption in the proximal tubule is reduced, leading to an increased sodium load delivered to the macula densa, thereby activating the tubuloglomerular feedback mechanism, resulting in afferent arteriolar constriction and a reduction in intraglomerular pressure (24). This hemodynamic effect is the core mechanism by which SGLT2 inhibitors reduce proteinuria and slow the decline in eGFR, and also explains the mild, reversible decrease in eGFR commonly observed at treatment initiation—a phenomenon analogous to the “initial hemodynamic effect” of RASi, indicating effective reduction of intraglomerular pressure (25).

From the perspective of hematuria, the reduction in intraglomerular pressure could theoretically alleviate mechanical stress injury to the glomerular capillary wall. In IgAN, the glomerular basement membrane and endothelial cell layer are rendered fragile by immune-mediated inflammation, and the hemodynamic pressure associated with hyperfiltration may further exacerbate capillary wall rupture and erythrocyte leakage. By reducing intraglomerular pressure, SGLT2 inhibitors could potentially decrease this mechanical erythrocyte leakage indirectly; however, this inference currently lacks direct clinical validation. The CREDENCE trial (27), DAPA-CKD trial (28), and EMPA-KIDNEY trial (29) all confirmed the kidney-protective effects of SGLT2 inhibitors in a broad CKD population, and the DIAMOND trial also provided evidence of proteinuria reduction in non-diabetic CKD (30); however, none of these trials included hematuria as a prespecified endpoint, and therefore the impact of hemodynamic effects on hematuria remains speculative. This hemodynamic pathway thus represents the only mechanism in this section that rests on direct clinical trial evidence in kidney disease populations; the anti-inflammatory and cytoprotective mechanisms discussed below are, by contrast, derived from preclinical and non-IgAN models and must be regarded as hypothetical as they apply to IgAN.

### 3.2 Anti-inflammatory and immunomodulatory mechanisms (extrapolated from non-IgAN models)

Beyond hemodynamic effects, preclinical and non-IgAN clinical studies suggest that SGLT2 inhibitors may exert anti-inflammatory actions, including suppression of the NLRP3 inflammasome and downregulation of the NF-κB pathway (26, 37, 38). In an ischemia-reperfusion model of kidney fibrosis, Ke et al. (39) reported that dapagliflozin modulated tubular fatty acid oxidation and the itaconate–NLRP3 axis, while Sim et al. (40) observed similar NLRP3 suppression in a diabetic context mediated partly through elevated ketone bodies. The extent to which these pathways operate in IgA nephropathy, in which glomerular injury is immune-complex-mediated rather than ischemic or metabolic, is presently unknown, and none of these findings has been replicated in patients with IgAN or in IgAN-specific experimental models.

**Figure 2:**
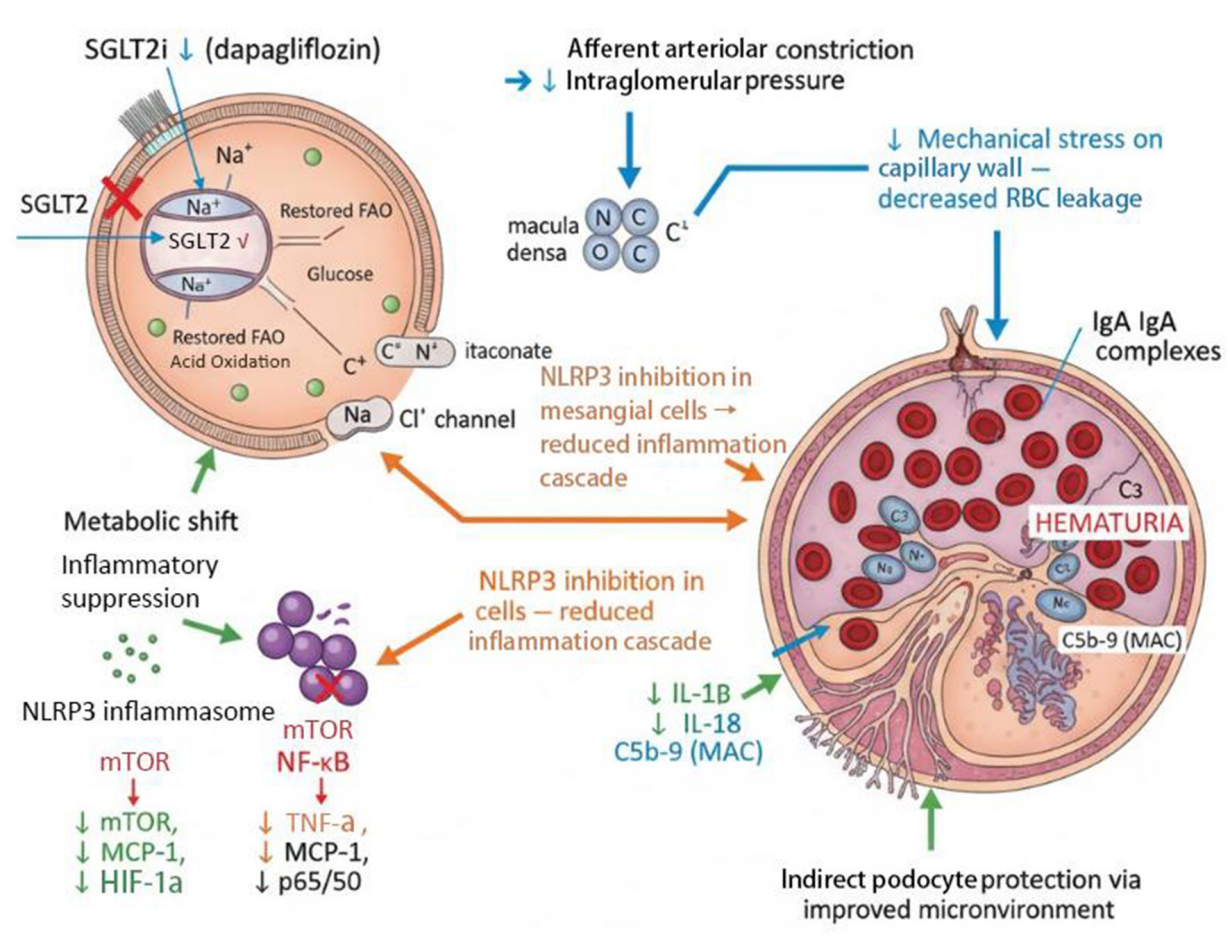
Proposed mechanisms by which SGLT2 inhibitors may reduce glomerular hematuria in IgA nephropathy. The tubuloglomerular feedback pathway is supported by clinical trial evidence. Anti-inflammatory mechanisms (NLRP3 inflammasome suppression, NF-κB downregulation, and indirect podocyte protection) are extrapolated from preclinical models of ischemia–reperfusion and diabetic kidney injury (26, 39, 40) and have not been validated in IgAN. FAO, fatty acid oxidation; MAC, membrane attack complex. Created with www.BioGDP.com

These data are therefore best regarded as generating a mechanistic hypothesis rather than establishing a mechanism: if, and only if, SGLT2 inhibitors attenuate intraglomerular inflammation in IgAN to an extent comparable to that observed in these non-IgAN models, it would be reasonable to propose that their influence on hematuria might exceed what hemodynamic effects alone could produce. This is an important caveat that should be kept in mind throughout the remainder of this review. (see also the explanatory note in Figure 2, which flags these pathways as extrapolated from non-IgAN preclinical models).

### 3.3 Potential effects on glomerular resident cells

In addition to hemodynamic and anti-inflammatory effects, SGLT2 inhibitors have been proposed to exert indirect protective effects on glomerular resident cells, primarily through reduction of oxidative stress and improvement of tubular cell energy metabolism (25, 26). In IgAN, mesangial cells are the principal target of immune-complex-mediated injury, and NLRP3 inflammasome activation in mesangial cells has been invoked to explain the amplification of inflammatory signaling in a “deposition–inflammation–proliferation” cycle (37). Whether SGLT2 inhibition can meaningfully interrupt this cycle in patients with IgAN, however, has not been directly demonstrated and remains conjectural.

A structural consideration further tempers expectations: SGLT2 is predominantly expressed in proximal tubular epithelial cells rather than in glomerular cells, so any effect on mesangial cells or podocytes must be indirect (24, 38). This implies that, even if the anti-inflammatory actions described above do occur in IgAN, the magnitude of their downstream effect on glomerular inflammation—and therefore on hematuria—is likely bounded by a ceiling: when the principal driver of glomerular injury is upstream immune complex deposition and complement activation, hemodynamic modulation combined with indirect anti-inflammatory effects may be insufficient to fully resolve the inflammatory process. This biological boundary is one of the reasons the comparative analysis in Section 5 contrasts SGLT2 inhibitors with agents that act closer to the pathogenic core of IgAN.

## 4 Clinical evidence: effect of SGLT2 inhibitors on hematuria in IgAN

### 4.1 Hematuria data from subgroup analyses of large randomized controlled trials

Clinical evidence for SGLT2 inhibitors in IgAN is primarily derived from subgroup analyses of large CKD trials; the principal studies and their hematuria-related reporting are summarized in Table 1. A prespecified IgAN subgroup analysis of the DAPA-CKD trial enrolled 270 patients and demonstrated that dapagliflozin significantly reduced the risk of the primary composite endpoint (sustained ≥50% decline in eGFR, ESKD, or cardiovascular or kidney death), with a clinically meaningful absolute risk difference (31). However, the endpoint design of this analysis was entirely centered on proteinuria and eGFR, and hematuria was not included as any prespecified or exploratory endpoint. Similarly, a secondary analysis of the EMPA-KIDNEY trial, which evaluated subgroups by primary kidney disease type, confirmed that the reduction in kidney progression risk with empagliflozin in the glomerular disease subgroup (including IgAN) was consistent with that of the overall population (32), but likewise did not report hematuria-related data.

**Table 1:** Summary of clinical studies reporting the effect of SGLT2 inhibitors on hematuria in IgAN.

Study	Design	Sample size	SGLT2i type/dose	Follow-up	Hematuria measurement	Baseline hematuria	Endpoint hematuria	*P* value	Proteinuria change	Major limitations
Dong et al. (34)	Prospective cohort, single-arm	93 (62 completed 6 months)	Dapagliflozin 5/10 mg (*n* = 90); canagliflozin 100 mg (*n* = 3)	3 months, 6 months	Urinary sediment RBC/HPF	12.3 (IQR 3.5–38.2)	3 months: 8.9 (IQR 2.6–28.8); 6 months: significant decline	3 months: 0.001; 6 months: 0.01	3 months: −22.9%; 6 months: −27.1%	Single-center, no control group, hematuria as secondary variable
Wheeler et al. (31) DAPA-CKD IgAN subgroup	Pre-specified RCT subgroup analysis	270	Dapagliflozin 10 mg	Median 2.1 years	Not reported	Not reported	Not reported	—	UACR reduction of 26% (vs. placebo)	Hematuria not included as endpoint
Judge et al. (32) EMPA-KIDNEY subgroup	RCT secondary analysis	853 (glomerular disease subgroup)	Empagliflozin 10 mg	Median 2 years	Not reported	Not reported	Not reported	—	Favorable trend	IgAN hematuria data not separately reported
Hammad et al. (41)	RCT (pilot)	50 (mixed glomerulonephritis)	Empagliflozin 25 mg	3 months	Not separately reported	—	—	—	Significant reduction (vs. placebo)	Non-IgAN-specific study, hematuria not separately analyzed

The absence of hematuria as an endpoint in both of these landmark trials was not coincidental. At the time of trial design, proteinuria had already been accepted by regulatory agencies as a surrogate endpoint in IgAN clinical trials, whereas hematuria had not achieved a comparable validated status (33). Coupled with the heterogeneity of hematuria measurement methods and the lack of a widely accepted remission definition, incorporating hematuria into the endpoint framework of multicenter international trials presented operational challenges. This design-level gap means that currently, direct data on the effect of SGLT2 inhibitors on hematuria in IgAN cannot be obtained from the highest-quality randomized controlled evidence. Reviews of the treatment standards for IgAN have also noted that the existing evidence evaluation system for supportive therapy is built almost entirely on proteinuria and eGFR (15).

### 4.2 Changes in hematuria in observational studies

The most direct evidence to date regarding the effect of SGLT2 inhibitors on hematuria in IgAN comes from a prospective cohort study conducted by Dong et al. (34) at Peking University First Hospital. This study enrolled 93 patients with biopsy-proven IgAN, all of whom had been receiving adequate RASi therapy with persistent proteinuria, and SGLT2 inhibitors were added to their regimen (90 patients received dapagliflozin, three received canagliflozin). At 3 months, median proteinuria decreased from a baseline of 1.32 (IQR 1.00–2.24) g/24 h to 1.07 (IQR 0.65–1.68) g/24h (a reduction of 22.9%, *P* <0.001), with a further reduction to 27.1% at 6 months (*P* <0.001). Notably, urinary sediment erythrocyte counts also showed a significant decline: from a baseline of 12.3 (IQR 3.5–38.2) RBC/HPF to 8.9 (IQR 2.6–28.8) RBC/HPF at 3 months (*P* = 0.001), with statistically significant improvement in hematuria also achieved at 6 months (*P* = 0.01). Subgroup analyses demonstrated that the antiproteinuric effect of SGLT2 inhibitors was not influenced by age, baseline eGFR, proteinuria level, immunosuppressant use, or a history of diabetes or hypertension.

This study is the only clinical study to date that has quantitatively reported the effect of SGLT2 inhibitors on hematuria in IgAN, and its value lies in providing “proof-of-concept” level evidence. However, this result must be interpreted with caution: hematuria was documented only as a secondary observational variable without prespecified statistical power calculations; the study employed a single-center, single-arm design without a control group; the sample size was limited (only 62 patients completed the 6-month follow-up); and whether the improvement in hematuria was independent of the reduction in proteinuria was not analyzed separately.

### 4.3 Indirect evidence and inferences

In addition to the direct evidence described above, several lines of indirect evidence from different perspectives support the hypothesis that SGLT2 inhibitors may affect hematuria in IgAN. Hammad et al. (41), in a pilot randomized controlled trial, evaluated the effect of empagliflozin 25 mg on non-diabetic glomerulonephritis (including IgAN, membranous nephropathy, focal segmental glomerulosclerosis, and lupus nephritis). The results showed that proteinuria was significantly lower in the empagliflozin group compared with the placebo group, with a trend toward stable kidney function, but hematuria was not reported as an independent endpoint. The significance of this study lies in confirming the safety and preliminary efficacy of SGLT2 inhibitors in immune-mediated glomerular diseases, laying the groundwork for further investigation in IgAN.

Lv et al. (42) employed a Mendelian randomization approach, utilizing publicly available genome-wide association study data, to explore the causal relationship between SGLT2 inhibitors and IgAN. This study found that dapagliflozin may act on IgAN through the LCN2 and AGER gene targets, and colocalization analyses supported the existence of shared causal variants between these target genes and IgAN susceptibility. Although this genetic epidemiological evidence does not directly involve hematuria, from a causal inference perspective it suggests that SGLT2 inhibitors and IgAN may be linked through mechanisms beyond hemodynamics, indirectly supporting the hypothesis that they may influence disease activity, including hematuria.

Regarding combination therapy, Schanz et al. (43) published the first real-world evidence on the efficacy of sparsentan in patients with IgAN already receiving SGLT2 inhibitors. This study demonstrated that the addition of sparsentan to stable SGLT2 inhibitor therapy resulted in a further 62% reduction in urine protein-to-creatinine ratio (UPCR) from baseline at 14 weeks. Although this study did not separately report hematuria data, its results suggest that additive effects may exist between SGLT2 inhibitors and other kidney-protective agents, providing a clinical framework for future assessment of the combined impact of such therapies on hematuria.

### 4.4 Limitations of the existing evidence

A comprehensive appraisal of the above evidence reveals systematic limitations in the available data on the effect of SGLT2 inhibitors on hematuria in IgAN. At the endpoint design level, none of the large RCTs included hematuria as a prespecified endpoint, and the only study that reported hematuria data (34) documented it merely as a secondary observational variable. At the measurement methodology level, Dong et al. used urinary sediment erythrocyte counts (RBC/HPF), while other studies did not even disclose whether hematuria-related data were collected, precluding meaningful cross-study comparisons. At the study design level, the direct evidence comes from only a single Chinese single-center cohort with a limited sample size and no control group, and its generalizability to other ethnicities and populations remains uncertain. Soliman et al. (44), in their position statement, also noted that further studies are needed to evaluate the effect of SGLT2 inhibitors on hematuria in patients with glomerulonephritis. In the most recent update on therapeutic options in IgAN, Lim et al. (45), while affirming the role of SGLT2 inhibitors as supportive therapy for IgAN, similarly lacked discussion of their effects on endpoints beyond proteinuria and eGFR, reflecting the prevailing neglect of the hematuria endpoint within the field.

Kamal et al. (46) similarly emphasized that no validated serum or urinary biomarker has yet been accepted for treatment monitoring in IgAN, and that proteinuria and eGFR remain the only recognized prognostic indicators. This broader absence of validated biomarkers—rather than a deficit specific to SGLT2 inhibitor trials—helps explain why hematuria endpoints remain unstandardized.

## 5 Hematuria outcomes with novel disease-modifying therapies: a comparative perspective

### 5.1 Anti-APRIL monoclonal antibodies

A proliferation-inducing ligand (APRIL), as a key upstream driver of Gd-IgA1 production in the pathogenesis of IgAN, has emerged as a central target for targeted therapy. Monoclonal antibodies against APRIL suppress the synthesis of pathogenic IgA1 at its source, theoretically offering the potential not only to reduce proteinuria but also to improve hematuria by attenuating immune complex-mediated glomerular inflammation.

Sibeprenlimab is a humanized IgG2 monoclonal antibody that selectively binds and neutralizes APRIL. The phase 2 ENVISION trial enrolled 155 patients with IgAN at high risk of progression and randomized them in a 1:1:1:1 ratio to sibeprenlimab 2 mg/kg, 4 mg/kg, 8 mg/kg, and placebo, administered as monthly intravenous infusions for 12 months. The results showed that 24-h urine protein-to-creatinine ratios decreased from baseline by 47.2%, 58.8%, and 62.0% in the three dose groups, respectively, compared with only 20.0% in the placebo group. Regarding eGFR, the placebo group experienced a decline of 7.4 ml/min/1.73 m^2^ at 12 months, while eGFR changes in the sibeprenlimab groups ranged from −2.7 to +0.2 ml/min/1.73 m^2^, demonstrating significant stabilization of kidney function. Serum Gd-IgA1 and total IgA levels decreased by approximately 65% in the 4 and 8 mg groups (47). Regarding hematuria, urinary sediment microscopy showed that hematuria levels declined in the sibeprenlimab treatment groups while remaining unchanged in the placebo group, which was interpreted as possibly reflecting a reduction in glomerular inflammation (48).

The phase 3 VISIONARY trial further expanded the sample size, randomizing 510 patients with IgAN to subcutaneous sibeprenlimab 400 mg or placebo every 4 weeks. The prespecified interim analysis demonstrated that at 9 months, the 24-h urine protein-to-creatinine ratio decreased by 50.2% from baseline in the sibeprenlimab group, while the placebo group showed a 2.1% increase, yielding a placebo-adjusted reduction of 51.2% (*P* <0.001). Serum APRIL and Gd-IgA1 levels decreased by 95.8 and 67.1%, respectively. This trial included hematuria as an exploratory endpoint, and notably, 39% of enrolled patients were already receiving SGLT2 inhibitors at baseline, suggesting that sibeprenlimab can provide additional benefit on top of SGLT2 inhibitor therapy (49).

Zigakibart is another humanized IgG4 monoclonal antibody targeting APRIL. Its phase 1/2 trial (ADU-CL-19) evaluated both intravenous and subcutaneous routes of administration in patients with IgAN. At week 100, proteinuria decreased by 60% from baseline, and eGFR remained stable. Similar to sibeprenlimab, a significant reduction in hematuria was also observed following zigakibart treatment, accompanied by rapid and sustained decreases in IgA, Gd-IgA1, and IgM levels, with only a mild reduction in IgG (50).

Atacicept, a TACI-Fc fusion protein, simultaneously antagonizes both APRIL and B-cell activating factor (BAFF). The results of the phase 3 ORIGIN trial demonstrated that atacicept achieved significant proteinuria reduction and eGFR preservation in patients with IgAN (51). An open-label extension study further confirmed the durability of these benefits (52). Although publicly available data on hematuria from the atacicept trials are relatively limited, its broad suppression of upstream immune pathways (simultaneously targeting both APRIL and BAFF) would theoretically be expected to produce hematuria improvement effects similar to those of sibeprenlimab and zigakibart.

### 5.2 Complement inhibitors

Complement system activation is a key effector mechanism in the fourth hit of the four-hit hypothesis of glomerular injury in IgAN. Iptacopan is an oral complement alternative pathway factor B inhibitor. Its phase 2 trial enrolled patients with IgAN in a randomized, double-blind, placebo-controlled study. The results showed that iptacopan significantly reduced proteinuria (placebo-adjusted reduction of 38.3%), elevated baseline membrane attack complex (sC5b-9) levels returned to normal following treatment, and the safety profile was favorable with no increase in infection risk (53). Subgroup analyses were conducted according to baseline hematuria severity, and the reduction in proteinuria was consistent across subgroups with different hematuria levels. In theory, complement inhibition could directly reduce inflammation-driven erythrocyte leakage by blocking membrane attack complex-mediated injury to the glomerular capillary wall; however, the quantitative effect of iptacopan on hematuria itself has not been separately reported.

### 5.3 Targeted-release budesonide and sparsentan

Nefecon (targeted-release budesonide) acts on Peyer's patches by releasing the drug in the terminal ileum, thereby suppressing mucosal immune-mediated Gd-IgA1 production. Ouyang et al. (54) reported data on the use of Nefecon in patients with IgAN and severe kidney impairment, confirming its feasibility in a broader patient population. Nefecon achieved significant proteinuria reduction and improvement in eGFR slope in the NefIgArd trial; however, systematic reporting of hematuria endpoints has been limited.

Sparsentan, a dual endothelin A receptor and angiotensin II receptor antagonist, represents a different therapeutic strategy. The 2-year results of the PROTECT trial demonstrated that sparsentan achieved a greater magnitude of proteinuria reduction compared with irbesartan, with a trend toward eGFR preservation (55). Sparsentan acts primarily through hemodynamic mechanisms, placing it at a level of action more comparable to that of SGLT2 inhibitors—both falling within the category of “supportive therapy/hemodynamic intervention”—and its direct effect on hematuria has similarly not been adequately reported.

### 5.4 Differential effects of supportive vs. disease-modifying therapies on hematuria

Before comparing hematuria outcomes across the drug classes reviewed above, several important caveats regarding evidence heterogeneity must be emphasized. The studies summarized in Sections 5.1–5.3 and in Section 4 differ substantially in design (randomized controlled trials vs. single-arm observational cohorts), in endpoint status for hematuria (pre-specified exploratory endpoints, *post hoc* observations, subgroup stratification variables, or not reported at all), in measurement method (urinary sediment erythrocyte counts at standardized intervals vs. descriptive reporting), and in trial maturity (completed phase 3 vs. early phase 2 or small cohorts). These differences preclude formal head-to-head comparison, and the observations that follow should therefore be read as an exploratory pattern rather than as graded evidence on a common scale.

With these caveats in mind, an apparent pattern can be described. Disease-modifying therapies targeting upstream immune pathways—anti-APRIL monoclonal antibodies (sibeprenlimab, zigakibart)—which suppress Gd-IgA1 production at its source, have reported reductions in hematuria in their respective clinical trials. In contrast, supportive therapies acting primarily through hemodynamic mechanisms—SGLT2 inhibitors and sparsentan—although similarly effective in reducing proteinuria, currently have limited and indirect evidence regarding their effects on hematuria.

If this pattern were to hold in prospective head-to-head studies, one interpretation would relate it to the level of the IgAN pathogenic cascade at which each drug class acts. Proteinuria can arise both from filtration barrier injury due to active inflammation and from increased structural permeability due to chronic sclerotic changes, and both disease-modifying and supportive therapies have been shown to reduce proteinuria. Hematuria, which is thought to track more closely with active inflammatory injury to the glomerular capillary wall (although, as noted in Section 2.3, it cannot reliably distinguish inflammatory from chronic lesions on its own), could in principle respond more selectively when the inflammatory driver itself is suppressed. Thompson et al. (56) noted that proteinuria reduction has been accepted as a surrogate endpoint for IgAN trials; the exploratory observation here is that drugs with similar magnitudes of proteinuria reduction might nevertheless differ in their effects on hematuria, if this difference survives formal testing. On that conditional basis, hematuria could plausibly serve as a supplementary indicator to help distinguish the “depth” of a treatment effect—symptomatic hemodynamic reduction vs. disease-modifying suppression of inflammation—but this proposal remains a hypothesis rather than a validated framework. Coppo (57), in an outlook on the future of IgAN treatment, also emphasized that more refined efficacy assessment tools are needed as the therapeutic repertoire expands.

Read across the “Study design/evidence level” and “Hematuria endpoint level” columns of Table 2, an apparent pattern emerges: agents acting on upstream immune pathways (anti-APRIL monoclonal antibodies) are the only class for which hematuria has been incorporated as a pre-specified endpoint in randomized trials, albeit exploratory; the complement inhibitor iptacopan used hematuria as a stratification variable without separately reporting change; and downstream hemodynamic agents (SGLT2 inhibitors, sparsentan) currently have either single-cohort observational data or no reported data on hematuria at all. This apparent gradient is compatible with—but does not demonstrate—the hypothesis that drugs acting closer to the pathogenic core of IgAN might produce more pronounced hematuria responses. The differences in underlying evidence quality across these drug classes are substantial, and the pattern must therefore be interpreted as hypothesis-generating rather than as a comparative ranking of biological effect.

**Table 2:** Cross-comparison of the effects of different drug classes on hematuria in IgAN.

Drug	Class	Therapeutic target	Study design/ evidence level	Proteinuria reduction	eGFR preservation	Hematuria change	Hematuria endpoint level	Key references
Sibeprenlimab	Anti-APRIL mAb	APRIL → ↓Gd-IgA1 production	Phase 2 RCT (ENVISION); Phase 3 RCT interim analysis (VISIONARY)	−47% to −62% (phase 2); −50.2% (phase 3)	eGFR stable	Urinary sediment erythrocyte decline (treatment group vs. no change in placebo)	Exploratory endpoint	Mathur et al. (47); Perkovic et al. (49)
Zigakibart	Anti-APRIL mAb	APRIL → ↓Gd-IgA1 production	Phase 1/2 open-label (ADU-CL-19)	−60% (week 100)	eGFR stable	Significant decline	Observational finding	Kooienga et al. (50)
Atacicept	TACI-Fc fusion protein	APRIL + BAFF → ↓B-cell activation	Phase 3 RCT (ORIGIN) + open-label extension	Significant reduction	eGFR preserved	Limited public data	Not separately reported	Lafayette et al. (51); Barratt et al. (52)
Iptacopan	Complement factor B inhibitor	Alternative complement pathway → ↓MAC formation	Phase 2 RCT	−38.3% (placebo-adjusted)	sC5b-9 normalization	Stratified analysis by baseline hematuria; change not separately reported	Subgroup stratification variable	Zhang et al. (53)
Nefecon	Targeted-release budesonide	Ileal Peyer's patches → ↓mucosal immunity	Phase 3 RCT (NefIgArd)	Significant reduction	eGFR slope improved	Limited reporting	Not systematically reported	Ouyang et al. (54)
Sparsentan	Dual ETA/AT1 antagonist	Endothelin + angiotensin → hemodynamic	Phase 3 RCT (PROTECT)	Superior to irbesartan (2 years)	Favorable trend	Not reported	Not included as endpoint	Rovin et al. (55)
SGLT2 inhibitors	SGLT2 inhibition	Tubuloglomerular feedback → hemodynamic + anti-inflammatory	Single-arm observational cohort (*n* = 62 completed; no IgAN-dedicated RCT)	−22.9% to −27.1%	eGFR preserved	3 months *P* = 0.001; 6 months *P* = 0.01 (single cohort)	Secondary observational variable	Dong et al. (34)

Entries in this table originate from studies of heterogeneous design (phase 3 randomized controlled trials to single-arm observational cohorts), differing endpoint status for hematuria, differing measurement methods, and differing trial maturity. The table is intended to display the current evidence landscape and the relative level at which each finding sits, not to imply that the findings are directly comparable on a common scale. Readers are referred to the “Study design / evidence level” and “Hematuria endpoint level” columns when interpreting any apparent between-class differences.

## 6 Clinical implications and future directions

### 6.1 Clinical implications and hypotheses for future evaluation

The analysis presented in this review suggests that, in addition to proteinuria and eGFR, it seems reasonable for clinicians to also record microscopic hematuria at follow-up visits during SGLT2 inhibitor therapy in patients with IgAN, given that it is a low-cost measurement. The role of proteinuria as the core indicator of treatment response in IgAN has been firmly established—a systematic review by Shah et al. (58) explicitly proposed the treatment principle of “lower proteinuria is better,” and KDIGO guidelines have set treatment targets of proteinuria <0.5 g/day or even <0.3 g/day (59, 60). However, a reduction in proteinuria may result either from a genuine alleviation of glomerular inflammation or merely from a reduction in filtration pressure attributable to hemodynamic effects. Concurrent observation of hematuria may, in principle, provide additional descriptive information that could help distinguish between these two scenarios, though whether it actually does so has not been formally tested.

It can be hypothesized—though this remains empirically untested—that if both proteinuria and hematuria decline concomitantly following SGLT2 inhibitor therapy, the treatment has attenuated both hemodynamic load and glomerular inflammatory activity; and conversely, that persistent or worsening hematuria despite proteinuria reduction might reflect residual immune-driven inflammation not fully controlled by SGLT2 inhibitors. Whether such a pattern should influence the decision to escalate to targeted immunotherapy (such as anti-APRIL monoclonal antibodies or complement inhibitors) is currently unknown, and we emphasize that this hypothesis should not, at present, guide treatment decisions outside a research setting. Floege et al. (23), in their call for a new paradigm in IgAN treatment, emphasized that future treatment strategies should simultaneously target both immune complex formation and glomerular hemodynamics; whether dynamic changes in hematuria can serve as a practical node for such multi-drug combination decisions is a question for future prospective study rather than a current management recommendation. The analogous use of urinalysis in monitoring protocols of other glomerular diseases—for example, in Fabry nephropathy (61)—illustrates that hematuria-based monitoring is methodologically feasible, but does not constitute evidence that it is yet appropriate for IgAN.

### 6.2 Need for standardization of hematuria measurement

A prerequisite for incorporating hematuria into the therapeutic monitoring of IgAN and clinical trial endpoints is the establishment of standardized measurement and reporting methods. Floege et al. (23), in their latest review, noted that hematuria detection methods vary significantly across different studies and clinical centers: dipstick testing detects hemoglobin rather than erythrocytes and cannot distinguish true hematuria from hemoglobinuria; manual urine sediment microscopy, while capable of directly counting erythrocytes, is subject to substantial variability due to specimen processing procedures and inter-observer subjectivity; automated urine analyzers can improve reproducibility, but cross-validation between different instrument platforms remains inadequate. This methodological heterogeneity is the principal barrier to cross-study comparison of hematuria data.

This review recommends that future IgAN-related research and clinical practice should prioritize automated urine sediment analysis as the standard method for hematuria detection to minimize human variability. Regarding remission definitions, reference may be made to the composite remission criteria proposed by Japanese investigators—urinary sediment erythrocytes <5/HPF sustained for at least 6 months, with a concurrent requirement of proteinuria <0.3 g/day. Zand et al. (21) also recommended the incorporation of unified hematuria assessment criteria into the screening and treatment monitoring of patients with IgAN. Only when consensus has been reached on both measurement methods and remission definitions can comparisons of the effects of different therapeutic agents on hematuria be considered reliable.

### 6.3 Future research directions

Based on the findings of this review, future research may advance in several directions. At the clinical trial design level, it is recommended that hematuria be incorporated as a prespecified secondary or exploratory endpoint in dedicated trials of SGLT2 inhibitors for IgAN. Data on the antiproteinuric effects of SGLT2 inhibitors are now relatively abundant; however, reliance on proteinuria alone may be insufficient to comprehensively evaluate the “depth” of treatment. As noted earlier (Section 4.4), the absence of validated biomarkers in IgAN (46) makes this gap particularly consequential—a situation that urgently needs to change.

At the efficacy assessment framework level, it is worth exploring the establishment of a composite evaluation framework integrating hematuria, proteinuria, and Gd-IgA1. The study by Canney et al. (62) demonstrated a dose-response relationship between the duration of proteinuria remission and prognosis in IgAN; Tang et al. (63) further revealed the dynamic association between time-varying proteinuria and disease progression; and Fang et al. (64) confirmed that achieving early proteinuria remission after treatment is closely associated with long-term outcomes. Incorporating the dynamic changes in hematuria into these time-dependent prognostic models may provide richer information on treatment response than single-time-point proteinuria assessment alone.

At the pathological validation level, the use of repeat kidney biopsies in patients with IgAN to correlate pre- and post-treatment changes in hematuria with histological alterations—particularly the E score and C score in the Oxford MEST-C classification—would help to confirm the biological basis of hematuria as a marker of disease activity from a pathological perspective. Floege et al. (23), in their proposed new paradigm, also emphasized that future treatment goals should extend beyond mere proteinuria control to normalization of the rate of kidney function decline (<1 ml/min/year), which requires more refined efficacy assessment tools. Hematuria, as a non-invasive, low-cost indicator that can be repeatedly measured at each follow-up visit, has the potential to become an important component of this refined assessment system.

## 7 Conclusion

This review has mapped the limited and largely indirect evidence bearing on the effect of SGLT2 inhibitors on microscopic hematuria in patients with IgAN. Direct evidence is extremely limited: to date, only one observational study has reported a significant reduction in hematuria following SGLT2 inhibitor therapy, while subgroup analyses of the IgAN cohorts from large randomized controlled trials (DAPA-CKD, EMPA-KIDNEY) did not include hematuria in their endpoint designs. From a mechanistic perspective, SGLT2 inhibitors offer plausible—but largely unvalidated in IgAN—pathways through which hematuria could theoretically be improved, including the reduction of intraglomerular pressure, suppression of the NLRP3 inflammasome and NF-κB pathway, and indirect protection of glomerular resident cells; however, because SGLT2 is expressed only in the proximal tubule, its intervention in glomerular inflammation is necessarily indirect, and the magnitude of improvement may have a ceiling. In contrast, anti-APRIL monoclonal antibodies, which target upstream immune pathways, have demonstrated significant improvement in hematuria in clinical trials. This apparent disparity, while hypothesis-generating, is derived from indirect comparisons across heterogeneous studies and should not be overinterpreted. Taken cautiously, it is broadly compatible with the idea that hematuria might serve as a supplementary indicator for differentiating the depth of “symptomatic treatment” vs. “disease-modifying treatment.”

These considerations suggest that monitoring hematuria alongside proteinuria during SGLT2 inhibitor therapy is reasonable and low-cost, and may generate useful observational data on residual inflammatory activity; however, whether hematuria dynamics should formally influence treatment decisions—including escalation to targeted therapy—requires prospective validation before being adopted as routine practice. In trial design, future studies should include hematuria as a prespecified endpoint and establish standardized measurement protocols and remission definitions. It must be emphasized, and should be read as the overarching conclusion of this review, that the effect of SGLT2 inhibitors on hematuria in IgAN currently remains strictly at the hypothesis-generating stage. Elevating hematuria from a neglected incidental indicator to a validated treatment response endpoint still depends on confirmatory evidence from prospective, multicenter clinical studies with hematuria as a pre-specified endpoint.
